# Correction: Méndez-Blanco et al. Stabilization of Hypoxia-Inducible Factors and BNIP3 Promoter Methylation Contribute to Acquired Sorafenib Resistance in Human Hepatocarcinoma Cells. *Cancers* 2019, *11*, 1984

**DOI:** 10.3390/cancers15235668

**Published:** 2023-11-30

**Authors:** Carolina Méndez-Blanco, Flavia Fondevila, Paula Fernández-Palanca, Andrés García-Palomo, Jos van Pelt, Chris Verslype, Javier González-Gallego, José L. Mauriz

**Affiliations:** 1 Institute of Biomedicine (IBIOMED), University of León, Campus of Vegazana s/n, 24071 León, Spain; 2 Centro de Investigación Biomédica en Red de Enfermedades Hepáticas y Digestivas (CIBERehd), Instituto de Salud Carlos III, Av. de Monforte de Lemos 5, 28029 Madrid, Spain; 3 Service of Oncology, Complejo Asistencial Universitario de León, Calle Altos de Nava, s/n, 24001 León, Spain; 4 Laboratory of Clinical Digestive Oncology, Department of Oncology, KU Leuven and University Hospitals Leuven and Leuven Cancer Institute (LKI), 3000 Leuven, Belgium

In the original publication [1], there was a mistake in Figure 2b as published. The confocal representative image of HepG2 control for HIF-2α (Figure 2b, right panel) was wrong due to an unintentional error in the selection during the edition process. The complete Figure 2 appears below. The authors apologize for any inconvenience caused and state that the scientific conclusions are unaffected. This correction was approved by the Academic Editor. The original publication has also been updated.

## Figures and Tables

**Figure 2:**
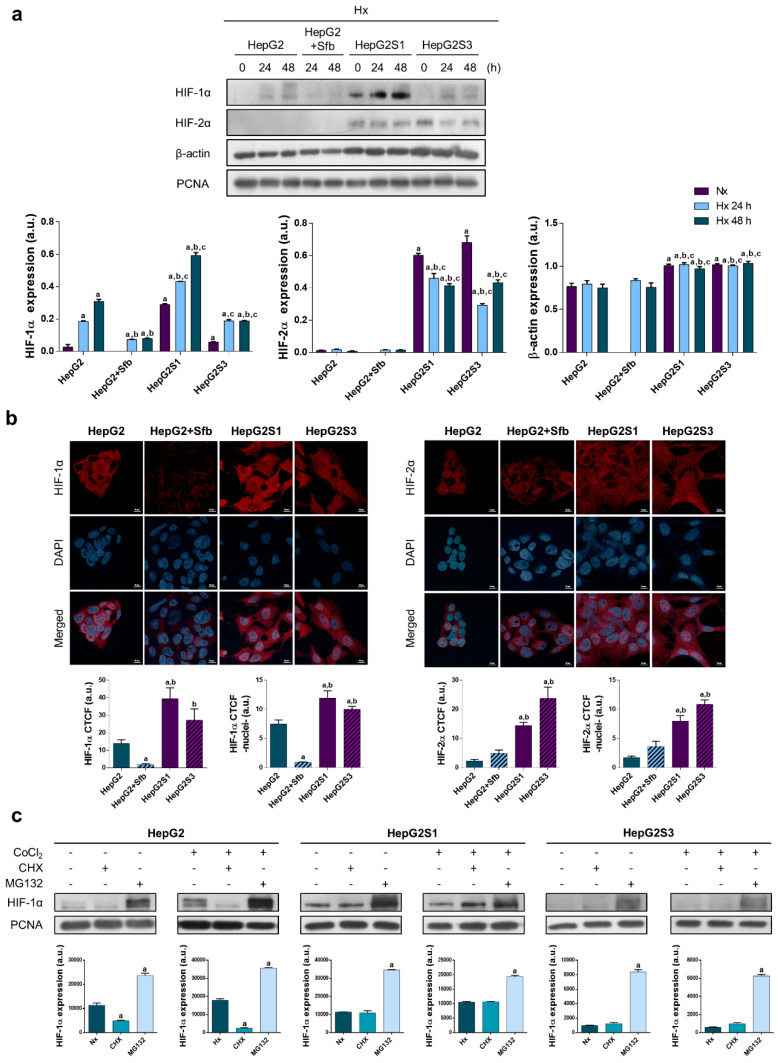
Cell modulation of hypoxia response in sorafenib resistance: (**a**) Effect of hypoxia on protein expression. Lanes 0 h show normoxic basal protein levels. ^a^ *p* < 0.05 vs. normoxic non-treated HepG2 cells, ^b^ *p* < 0.05 and ^c^ *p* < 0.05 vs. hypoxic non-treated and sorafenib-treated HepG2 cells, respectively, at each time point; (**b**) Confocal images of hypoxia-inducible factor (HIF)-1α (left panel) and HIF-2α (right panel) immunofluorescence staining (red) show HIFs expression after incubation under hypoxia for 24 h. DAPI staining (blue) denotes cell nucleus. Magnification: 63×, scale bar: 10 µm. Bar graphs at left position represent total expression whereas bar graphs at right position represent nuclear translocation for each marker analyzed. ^a^ *p* < 0.05 and ^b^ *p* < 0.05 vs. non-treated and sorafenib-treated HepG2 cells, respectively; (**c**) Evaluation of HIF-1α protein synthesis and degradation processes. ^a^ *p* < 0.05 vs. normoxia/hypoxia within the same cell type. Data from (**a**–**c**) are expressed as mean values of arbitrary units (a.u.) ± SD of three independent experiments. Full-length immunoblots are presented in Supplementary Figure S1a.
