# Supplementary material for: Stabilization of Hypoxia-Inducible Factors and BNIP3 Promoter Methylation Contribute to Acquired Sorafenib Resistance in Human Hepatocarcinoma Cells
Source: Cancers (Basel). 2019 Dec 9;11(12):1984. doi: 10.3390/cancers11121984 (PMC6966438; doi:10.3390/cancers11121984)
Supplement: Supplementary file 1 [file cancers-11-01984-s001.pdf]

Supplementary Materials

Stabilization of Hypoxia-Inducible Factors and BNIP3 Promoter Methylation Contribute to Acquired Sorafenib Resistance in Human Hepatocarcinoma Cells

Carolina Méndez-Blanco, Flavia Fondevila, Paula Fernández-Palanca, Andrés García-Palomo, Jos van Pelt, Chris Verslype, Javier González-Gallego and José L. Mauriz

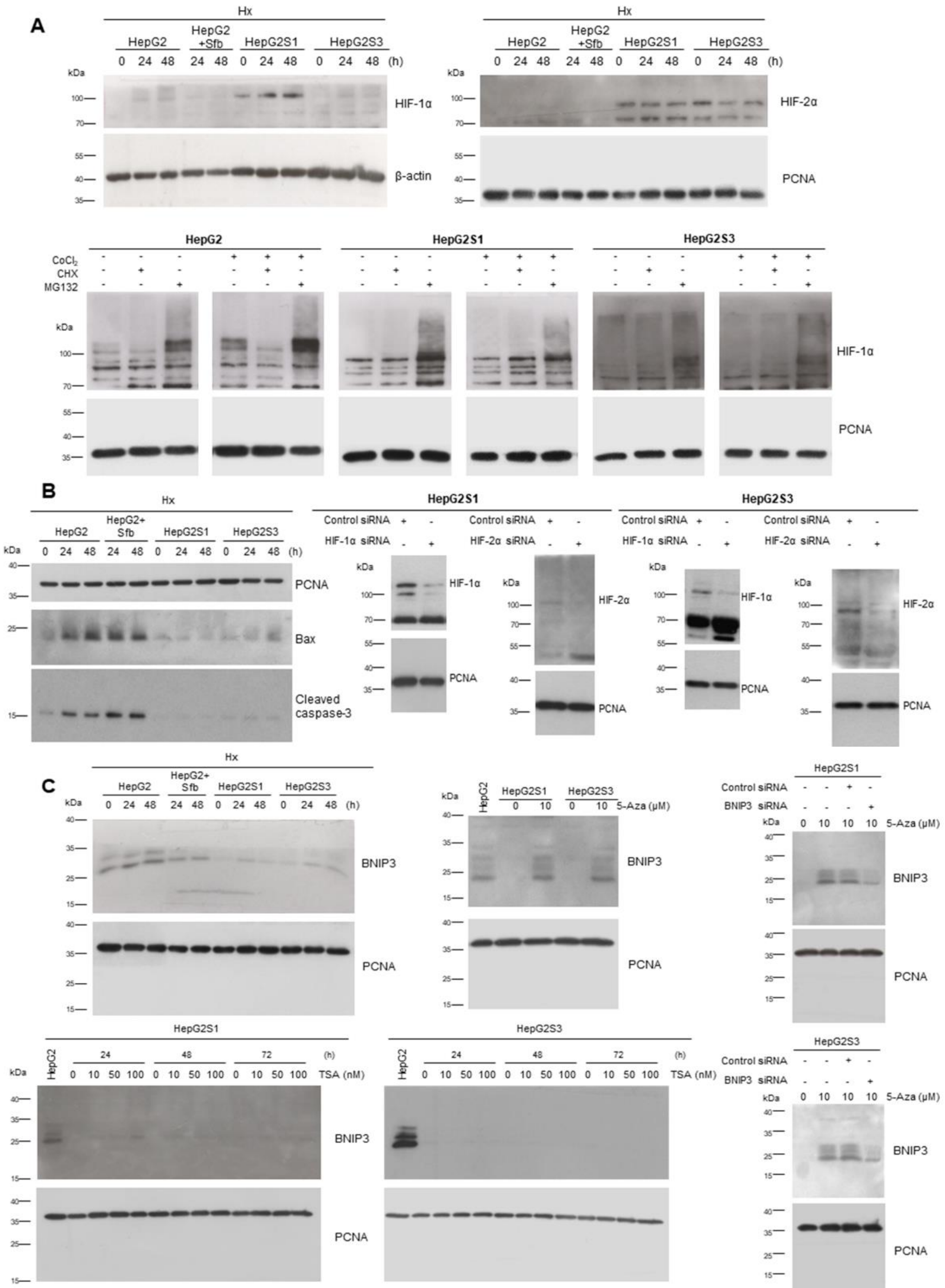

Figure S1. Full-length immunoblot images from Figures 2, 3 and 4: (**a**) Complete Western blot images of HIF-1α, HIF-2α, β-actin and PCNA from Figure 2 are represented. HIF-2α and PCNA were reincubated after stripping in the blots of HIF-1α and β-actin from Figure 2a, respectively; (**b**) Full Western blot images of Bax, cleaved caspase-3 and PCNA from Figure 3b and immunoblots of HIF-1α, HIF-2α and PCNA from Figure 3c; (**c**) Total BNIP3 and PCNA immunoblots from Figure 4. PCNA were reincubated after stripping of BNIP3 blots. PVDF membranes from (**a**) and (**b**) were cut to incubate with different antibodies simultaneously.

© 2019 by the authors. Licensee MDPI, Basel, Switzerland. This article is an open access article distributed under the terms and conditions of the Creative Commons Attribution (CC BY) license (http://creativecommons.org/licenses/by/4.0/).
